# Analysis of consumer product preference and news media based on data mining technology

**DOI:** 10.3389/fpsyg.2022.1007846

**Published:** 2022-09-21

**Authors:** Fang Wang, Zengguang Fan, Yuhui Qi

**Affiliations:** ^1^School of Journalism and Communication, Zhengzhou University, Zhengzhou, Henan, China; ^2^Department of Psychology and Behavioral Sciences, Zhejiang University, Hangzhou, Zhejiang, China; ^3^School of Education, Zhengzhou University, Zhengzhou, Henan, China

**Keywords:** field marketing, news dissemination, media analysis, media communication, product preferences

## Abstract

In order not to be eliminated by the market, enterprises must face various consumer preferences, design products that meet consumer preferences, and enhance competitiveness. This paper combines on-the-spot marketing to study the product preferences of consumers and the personality characteristics of media hosts. This paper introduces the data mining technology of news media into the research of consumer’s preference for products. Based on the comprehensive use of media mining technology, customer research theory, and product background and foundation, the specific process of influencing consumers’ product preference is established. It can be seen from the study that the personality of the anchor has a great relationship with the consumption level of consumers and the sales rate of products, with an impact of 78.53%. Through this study, we can see that there is a certain relationship between consumers’ product preference and anchoring personality. Studying the basic characteristics of the phenomenon live broadcast commodity marketing model has important theoretical value for analyzing the live broadcast commodity marketing model. It points out the direction for the scientific, healthy, and sustainable development of the future live broadcast commodity marketing mode.

## Introduction

The news media has the characteristics of “fragility,” not only because of the timeliness of news content. And now the audience has many information channels, and the pace of life and environment change rapidly. Many other contents in the media will soon become known to the audience or have become obsolete. Therefore, the preservation value of news media is small, and the cost can be reduced by sacrificing its preservation. For example, newspapers use cheap paper and are simply folded without binding. Therefore, the news media are “fast food cultural goods,” which are discarded as soon as people use them. Some articles or documents are published in newspapers. It can also be published in well-preserved periodicals, anthologies, or pamphlets, especially when there are many media and everyone can access only a small part of them. Relativity means that news media have different values for different consumers (Xiong et al., 2021). A certain kind of clothing can keep everyone warm or decorate, a certain kind of food can satisfy everyone’s hunger or taste, and the news media has different use values for different consumers. Some news will be useful to some people, but not at all to others. It has the function of knowing for some people, but only entertainment for others. Therefore, the dissemination of news media should be very clear and understand their target audience, and strengthen pertinence. Consumers always choose and use media to meet their own needs. In the “buyer’s market” with fierce media competition. The news media must provide satisfactory communication services according to the audience’s motivation, needs, attitude, personality, psychology, etc. Sometimes, through inspiration and guidance, the audience should be aware of their own interests and needs and the corresponding services of the media, so that the audience can fully recognize and have a sense of satisfaction, and form a preference for the choice of relevant media. The needs of consumers are not only practical, but also spiritual (Tsfati et al., 2020). The audience generally regards the news media as not only an information source, but also a cultural and entertainment product, and even a daily spiritual sustenance. The higher people’s living standards, the more prominent their spiritual needs. Now creative economy and experience economy are increasingly valued, which is related to this. The essence of this kind of creativity is to bring new satisfaction, new functions, new convenience, or new spiritual satisfaction, rather than strengthening and improving the original functions (Cosgrove et al., 2019). It was first proposed that making mobile phones into larger capacity and more sensitive signals was an improvement idea, and it was first proposed that making mobile phones for women, lovers, etc., preferred to be creative. The experience economy focuses on the consumption process, so that people can experience and enjoy the feeling worthy of experience (Hopp et al., 2020). It is precisely because people’s living standards have reached a certain height that many spiritual needs that were overwhelmed by material needs in the past have been released. The creative economy and experience economy have become important economic phenomena by providing a wide range of opportunities for the creative economy and experience economy. People of insight advocate that the news media is a creative product, and the news media industry is one of the creative industries (Dergaliuk et al., 2021). To develop creative economy and experience economy, it is necessary to improve humanistic quality, pay attention to spiritual care, and encourage personalized characteristics and creative thinking, especially for the news media (Rosyadi et al., 2020).

The communication between things and people is transmitted through the function and form of news media. When designers create products, they also give products a certain form. People will get all kinds of information in the process of using products, which will cause different emotions. Nowadays, consumers regard personal emotional needs as the focus of their choice and purchase decisions, which has also brought great changes in consumption patterns (Kim and Sullivan, 2019). Sometimes when consumers buy a certain bathroom product, they not only want to obtain a certain function of the product, but also want to express their value proposition and show their lifestyle through the brand. If the enterprise ignores this point in brand positioning and blindly emphasizes the attributes and functions of the product. If they cannot meet the more psychological needs of consumers, they will be gradually eliminated by the market. The emotional appeal carried by products is enough to affect consumers’ preferences for products. In the past, design and manufacturing behavior was based on the unilateral behavior of designers. They considered the production technology or cost-effectiveness level, and even developed new products according to the subjective opinions of company leaders or designers, without considering consumer preferences. Now, consumers have got rid of the old habit of focusing only on product functions, and the stimulation of demand is linked to the taste of product design. Emotional consumers often like novel, unusual, and emotional designs, which can provide consumers with more imagination (Zollo, 2021). Information is anything that can be digitized (encoded into bytes), such as books, databases, movies, music, stock indexes, software, etc. All these are information products. There are many differences between the production of information products and that of material products, which makes information products have significantly different economic characteristics from material products. Information products have the economic characteristics of high development cost, low reproduction cost, consumption scale effect, public goods, and so on. Therefore, marginal cost pricing or market demand pricing is not applicable to the pricing of information products. The most feasible strategy is to price according to the value of information products to different consumers, and the value of information products largely depends on consumers’ preferences. This paper uses a variety of research methods to analyze and study it. In consumer research, the corresponding model diagram and algorithm formula are established for analysis and research. In the research of marketing and host personality characteristics, the corresponding data chart is established to analyze and study it.

The contribution of research innovation lies in the research and analysis of product consumption using news media mining technology. Based on consumers’ preference for products, this paper analyzes the characteristics of news media communication. This paper implements a consumer behavior analysis system based on data mining. Data mining technology is applied to consumer behavior analysis in customer relationship management. It mainly realizes the three mining objectives of consumption behavior analysis, live broadcast consumption behavior analysis and consumption satisfaction analysis. The system has friendly interface and convenient operation, which provides a convenient way for schools to understand the consumption status of students and teaching staff.

## Related work

One of the consequences of the technological revolution with information technology as its core and its wide application is the mass and diversified ways of knowledge and information dissemination. Faced with a huge amount of information, consumers have more active choices while being overwhelmed (Wang et al., 2021). In the traditional mode of communication, mass media, such as television, radio, newspapers, and magazines, as the disseminators of information, control the contents, ways, and channels of information dissemination, and become the main controller of information, while the public can only exist as receivers of information and play a completely passive role. The early communication theories, such as “Bullet Theory” and “Subcutaneous Injection Theory,” reflect the audience’s status (Saragih and Harahap, 2020). The communication revolution accompanying the information technology revolution has led to the change of communication mode and the change of audience role. Live delivery refers to the marketing mode of selling goods live through the live video platform. At present, Taobao, Auto Quicker, and TikTok are the main platforms with goods, and JD.COM, Pinduoduo, Youzan, and other e-commerce platforms have also begun to test the game of live broadcast with goods (Lee et al., 2018). The rapid development of science and technology not only makes the Internet of everything possible, but also gives birth to new business models such as live broadcast and goods delivery. The explosive development of live broadcast business model in China is closely related to the worldwide spread of COVID-19 epidemic since 2020. In recent years, the product categories of live broadcast with goods have been increasing day by day, especially since the COVID-19 epidemic (Chen et al., 2021). From daily consumer goods to launch services of launch vehicles, goods are carried in live broadcast rooms, and there is a great trend that everything can be carried life. Some large-scale international trade fairs, new technology development conferences, and new product launches also bring new opportunities for the vigorous development of live broadcast goods. Under the network marketing mode, consumers gathered in the homogeneous space have the characteristics of high convergence and similarity, which makes the role of personal factors and social factors no longer obvious. However, the marketing role of platform anchors as carriers is more important, and it is more necessary to re-examine consumer behavior from the perspective of platform marketers (Yan, 2018).

Human basic needs are divided into five levels, namely physiological needs, safety needs, belonging and love needs, respect needs, and self-realization needs. Among them, the physiological demand is the lowest level demand, the five demands increase in turn, and the self-realization demand is the highest level demand. Zhang L believes that the emergence of demand levels at all levels is closely related to individual development. There are all kinds of basic needs in different periods, but in each different development stage, the intensity of all kinds of basic needs is different, among which the one with the greatest intensity is called the dominant demand, and those with other intensities in a secondary position become the secondary demand (Ahmad, 2019). Consumers’ decisions depend on relevant commodity information. However, irrational views, including psychoanalysis, social activities, impulsiveness, and random selection, all emphasize that purchasing activities meet emotional and non-physical needs, and think that consumers lack due information about most products, and are often reluctant to spend energy comparing and evaluating goods (Petrescu et al., 2020). This view denies that all consumption behaviors are conscious and rational, and denies that consumers are completely rational consumers who pursue the maximization of interests. CRAIG DILOUIE believes that the result of consumers’ purchase decision is reflected in the satisfaction consumers get through this commodity, that is, the enjoyment it provides and the feeling that it ultimately makes consumers feel satisfied (Anica-Popa et al., 2021). Consumers’ attention to feelings is not only reflected in the consumption process of products, but also the advertising psychology that consumers are eager to get a good feeling and experience from advertisements has already been keenly discovered by advertising creators. Meeting the entertainment needs of consumers requires that advertisements bring entertainment experiences such as fun, sensory enjoyment, emotional experience, and good feeling to the audience. Hsu WY believes that the focus of customer relationship management is to automate and improve business processes related to customer relationships in areas such as sales, marketing, customer service, and support (Hwang and Lee, 2022). Customer relationship management is not only a set of principles and systems, but also a set of software and technology. Its goal is to shorten the sales cycle, reduce sales costs, increase revenue, find new markets and channels to expand business, and improve customer value, satisfaction, profitability, and loyalty. The phenomenal on-site delivery marketing model focuses on the matching of the basic attributes of delivered goods with the anchor, the combination of delivery style and professional quality, and the integration of commercial marketing teams and brand manufacturers, so as to achieve accuracy, reliability, and fluency of live broadcast marketing, and finally improve the delivery effect of live broadcast (Siagian et al., 2020).

## Viewing news media from consumer products

### Research on consumer goods preference based on media data mining

**Figure 1:**
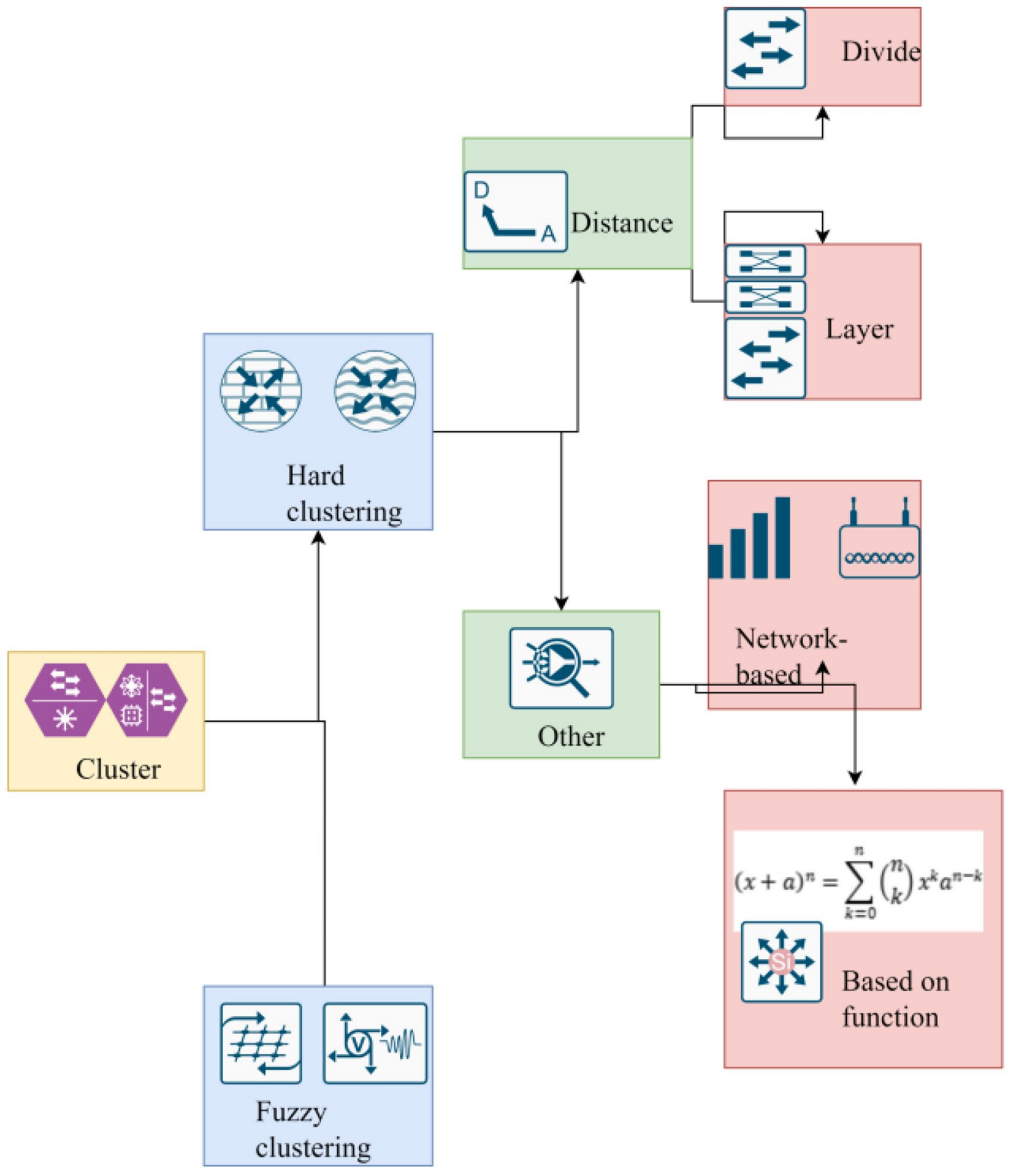
Grid based method and model-based method.

Media consumption preference refers to consumers’ enthusiasm for a certain media or the content of a certain aspect of a certain media. Even have a special love for a column or a program host, etc. This is the ranking of the media available for consumption by consumers, which is related to personal economy, knowledge, hobbies, energy, etc. Welfare economists assume that people are rational, and they will try to meet their preferences for goods and services within the scope of income, which is also known as explicit preference. This theory also plays a role in media consumption. Most media supported by advertisements do not charge consumers directly. Thus, consumers’ preferences can be freely expressed from the choice of programs. Because the marketing activities of enterprises are centered on meeting the market demand, the satisfaction of the market demand can only be achieved by providing certain products or services. In marketing, to develop new products or innovate products, we can start with consumers’ potential needs, develop new products that meet consumers’ potential needs by discovering and digging them, or improve and innovate existing products to find new product growth points (Ebrahim, 2020). The important role in the development of information organizations is increasingly recognized by people, and many organizations have developed various information collection and processing systems. These systems not only bring the convenience of information processing to the organization, but also bring precious wealth-a large amount of precious data to the organization. There are extremely important business knowledge hidden behind these data, but these business knowledge are implicit, unknown in advance and potentially useful. Applying data mining technology to consumer behavior is mainly applied to the analysis layer. Processing existing information, such as consumption behavior analysis, is to provide the ability to analyze customer consumption data and consumer behavior patterns. Provide support for the marketing strategy decision of enterprises. Only the CRM system using data mining technology is an efficient CRM system. Because of its own characteristics and functions, data mining will play an increasingly important role in customer relationship management system. Data mining involves many disciplines and methods. According to mining tasks, it can be divided into classification or prediction model discovery, data summarization, clustering, association rule discovery, sequential pattern discovery, dependency or dependency model discovery, anomaly, and trend discovery, etc. According to mining methods, it can be divided into machine learning methods, statistical methods, neural network methods, and database methods. Cluster analysis originated from taxonomy, which used to be classified by its own experience and professional knowledge. With the emergence and accumulation of huge data sets, the classification method based on individual ability is no longer applicable, resulting in the real clustering method (Jing et al., 2019). As a common method in clustering data mining, it can help people to process data without any information, thus generating category information. The core idea of clustering can be summarized as “like attracts, while opposite attracts.” In the research of clustering method, a corresponding model diagram is established to analyze and study it, as shown in Figure 1.

**Figure 2:**
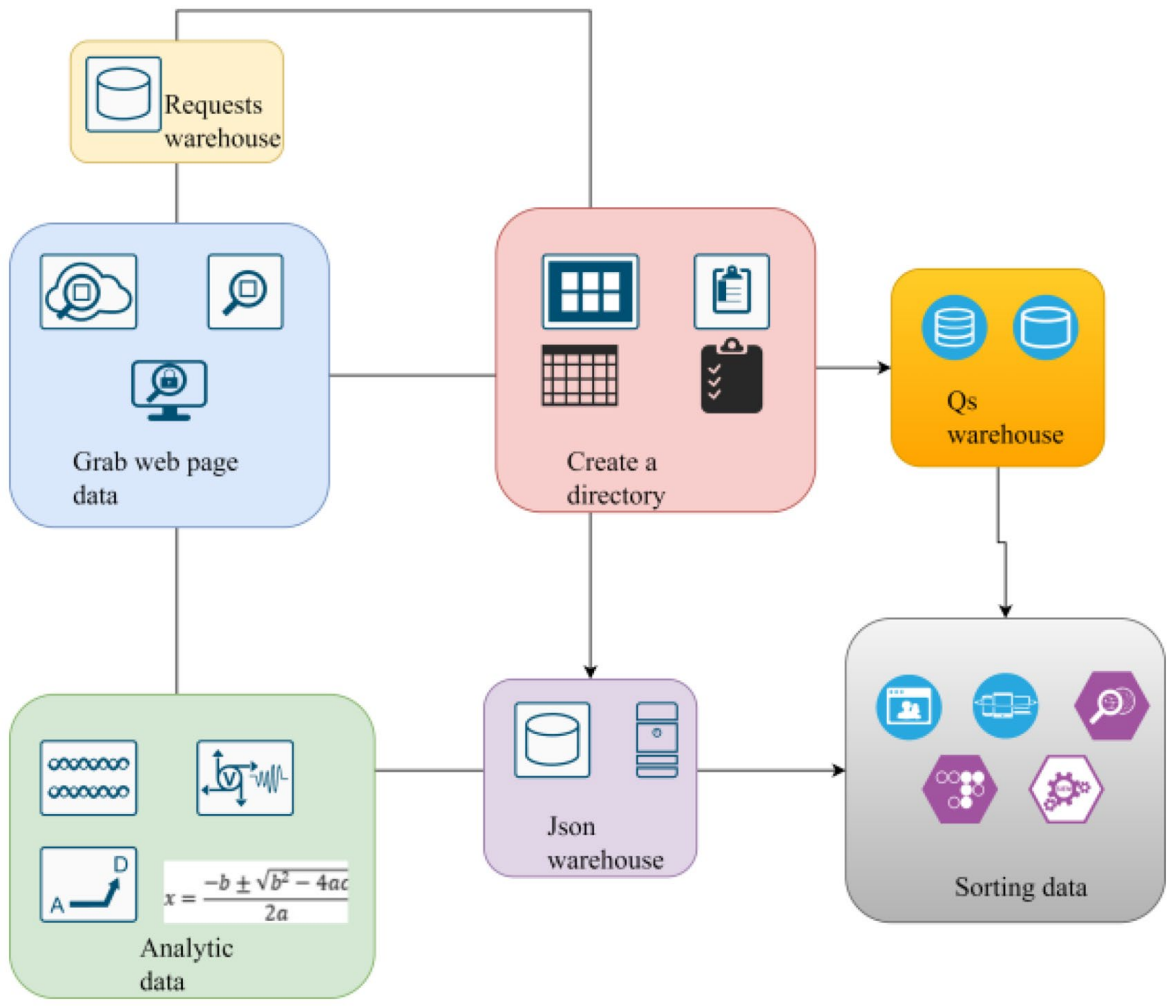
The data crawling flow chart.

This study adopts some news media comment mining methods, including product feature analysis and emotional consumer comment analysis. The study of consumer comments can provide a reference for consumers to choose goods. Provide ideas for businesses to improve the quality of goods and services, as well as platform methods to improve communication and logistics services between businesses and consumers. Collect, clean up, and mine news comment data, use word frequency and word cloud analysis to extract product features, and understand consumers’ preferences for product attributes. And use the methods of calculating consumers’ emotional tendency to explore consumers’ emotional tendency and its degree. Combined with the results of the above analysis methods, we further study the relationship between consumers’ preferences for product attributes and consumers’ emotional tendencies. In the study, the corresponding model diagram is established for analysis, as shown in Figure 2.

**Figure 3:**
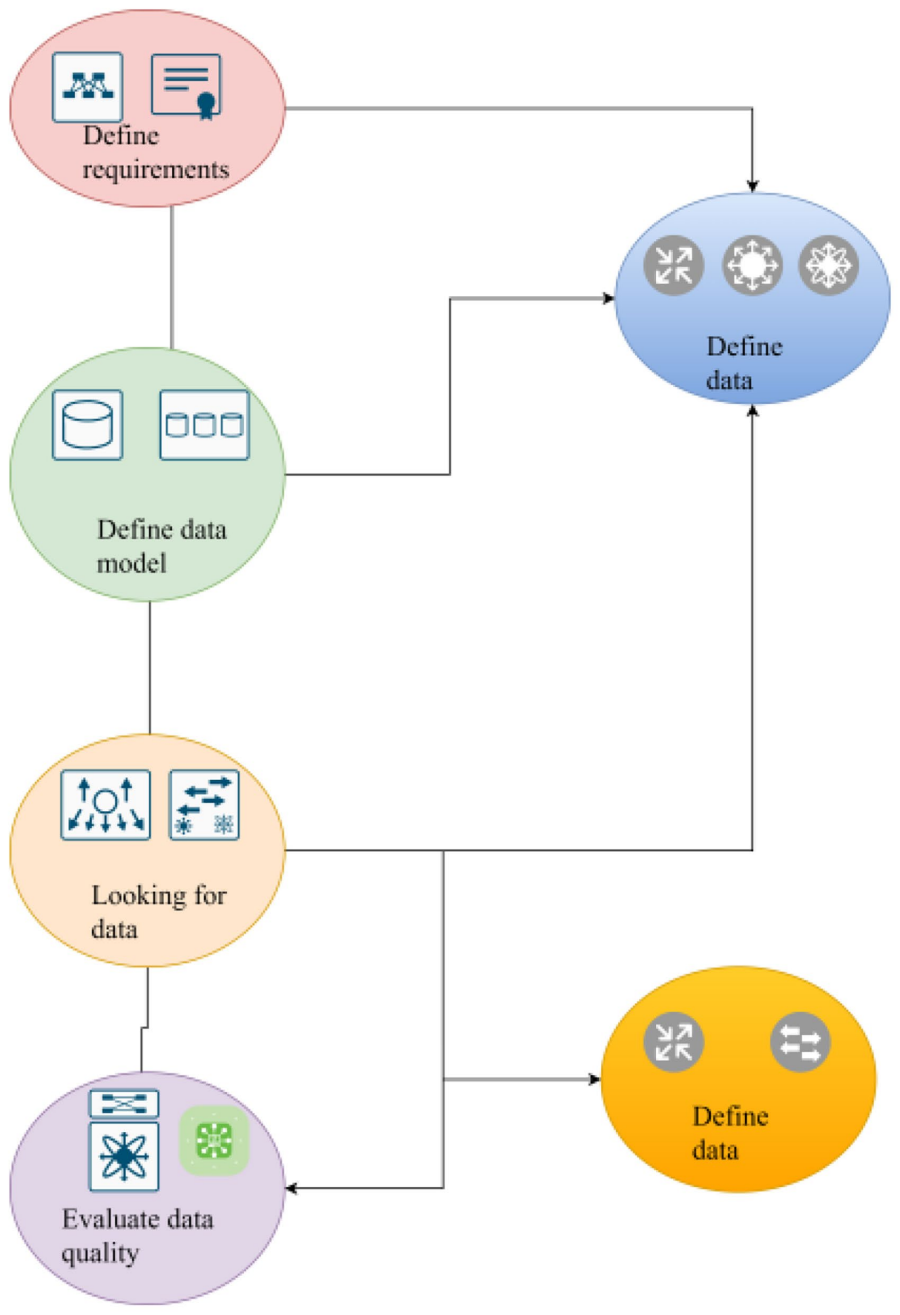
Information content in data mining tools.

The content of consumer behavior analysis is mainly to analyze various factors that affect consumer psychology and consumer behavior. Analyze various consumer psychology and behavior phenomena, and reveal the development and change laws of consumer behavior. Analysis of consumer demand and motivation psychology research shows that the starting point and motive force of human behavior is human demand. If you want to analyze and understand the consumer behavior, you must first analyze and study the needs of consumers. Demand is the motive that produces, and the motive guides the behavior, which ultimately affects demand. Therefore, it is analyzed and studied by establishing a general mining method, as shown in Figure 3.

Cluster analysis is a multivariate statistical analysis method to study “birds of a feather flock together.” The basic idea of clustering analysis is to aggregate categories according to the correlation between objects. Before cluster analysis, these categories are hidden, and it is unknown in advance how many categories they can be divided into. The principle of clustering analysis is that individuals in the same category have great similarity, but there are great differences between different categories. Variable clustering is also called type clustering. There are many variables that reflect the characteristics of the same thing, and some variables are often selected to study a certain aspect of the thing according to the problem under study. Because human beings have limited knowledge of objective things, it is often difficult to find representative variables that are independent of each other, thus affecting further understanding and research of problems. An important purpose of principal component analysis is also to make a comprehensive evaluation of the original variables. Each principal component is a linear combination of original variables, representing some essential attributes hidden behind the original variables. Cluster analysis is a method to study individual classification according to the characteristics of things. The principle of cluster analysis is that the individuals in the same class have great similarity, and the individuals in different classes have great differences. At the beginning of clustering, each sample or variable in a sample is a class of its own. By calculating the similarity measure between samples or variables, the two most similar samples or variables are merged. After merging, the number of classes is reduced by recalculating the similarity measure between classes, and then the two most similar classes are selected for merging. This process of calculation and merging is repeated until all samples or variables are grouped into one class, as shown in Table 1.

**Table 1:** Cluster analysis sample data table.

	Indicator 1	Indicator 2	..	Indicator n
Unit 1	X10	X13	..	X2P
Unit 2	X20	X23	..	X1P
Unit n	Xn1	Xn2	..	qqXnP

The core of the method is to divide the input observations directly according to certain principles and methods, and then improve the quality of clustering division by moving the objects in the division until the established division criteria are met. This method requires the number k of clustering results to be determined in advance, only in small-scale data, and the effect of finding spherical classes is better, but for non-convex classes. At present, there are two main methods used in emotion tendency analysis: the method based on machine learning and the method based on semantic dictionary: the method based on machine learning uses various classification methods of machine learning to identify emotions; However, the method based on semantic dictionary first constructs a dictionary or list of emotional words, and judges the emotional tendency with the help of this dictionary. Emotional inclination refers to the intensity of the subject when expressing positive or negative feelings toward the object. Different emotional degrees are often reflected by different emotional words or emotional mood. Usually, in order to distinguish the degree of difference between the two, different weights are given to each emotion word in the research of emotion inclination analysis. There are two kinds of affective tendency analysis methods: one is based on affective dictionary. One is based on machine learning, such as machine learning based on large-scale corpus. The method based on machine learning needs a large number of manually labeled corpora as training sets. By extracting text features and constructing classifiers, emotion classification can be realized. Therefore, the products are analyzed through examples, as shown in Table 2.

**Table 2:** Frequency of emotional tendency of product reviews.

Product	Favorable rate (%)	Bad review rate (%)
Mobile	81.23	18.31
Clothes	95.36	2.98
Furniture	89.31	8.97
Medicines and chemical reagents	87.31	15.98

Media quality refers to the combination of the intrinsic value, quality, and service level of a media. The stability and structure of quality is a relatively long accumulation process. Once formed, it rarely changes at will. It is on this point that the media has won the trust of consumers. Compared with the media without quality, consumers are more inclined to consume those high-quality media. Quality preference is formed over a long period of time, and once formed, it is difficult to change. Change means paying not only monetary costs, but also cognitive and emotional costs. For example, people’s preference for the quality of Spielberg films.

For different products, consumers pay attention to different product features. Consumers pay attention to many features: mobile phone’s running speed, camera effect, appearance, brand, screen, sound effects, and standby time all affect consumers’ satisfaction with products. Among them, the running speed and camera effect of electronic products such as mobile phones are consumers’ most concerned product features, and the appearance is consumers’ most concerned product feature. The product characteristics that consumers pay attention to under different platforms are different. Therefore, merchants should timely adjust the product introduction information in combination with the platform characteristics to meet the information needs of consumers on different platforms. Although consumers’ attention to different features of products mainly depends on the functions of different products, for any product, quality and cost performance are the focus of users’ attention. Therefore, consumers will analyze and choose the relevance of products to some extent, as shown in Table 3.

**Table 3:** Correlation analysis data table.

Product description quantity	Total comments	Bad review rate (%)
2	747	6.53
6	928	9.53
14	674	16.53
12	636	13.63

The more attributes consumers pay attention to, the higher the rate of bad reviews will be. The more consumers pay attention to different dimensions of a product, the more likely they are to be dissatisfied with the whole product due to dissatisfaction with one or some of its attributes. For example, for a certain mobile phone, a consumer pays attention to the features such as running speed, appearance, memory, and sound quality. If he is satisfied with the appearance, memory, and sound quality of the mobile phone, dissatisfaction with the running speed will also lead to dissatisfaction with the whole product. For different products, it is necessary to improve the products according to the product characteristics that consumers pay attention to. For example, if consumers pay attention to the running speed of mobile phones, R&D departments should pay attention to the improvement of mobile phone fluency. The quality and packaging of books are the focus of consumers’ attention, so better quality paper can be used in printing, and books can be wrapped in mail to reduce the possibility of books being damaged in transit. Drugs are urgently needed supplies, so logistics management can be improved. In the actual selection process, due to the price and other reasons, people have to comprehensively consider multiple features of products, and often have to sacrifice some other features on the premise of meeting some requirements, which is a trade-off and compromise of features. Through joint analysis, we can simulate people’s choice behavior and predict the results of different types of people’s choices. Therefore, through joint analysis, we can know how much consumers attach importance to the features of products, and use this information to develop competitive products.

### Research on consumer product preference based on news media data mining

From the actual needs and preferences of the target consumer group, the media with different media attributes will be matched with the consumer’s cognition, understanding, and touch purchase decision-making process. For example, when consumers recognize an interesting product, they think that the information transmitted by the media with attractive, rich, and diverse forms of expression can be attractive. Image is related to vision, psychology, users’ life experience, and cultural background, and belongs to the category of psychological activities. Product image refers to consumers’ intuitive association with the form of products by virtue of their own senses, and among these associations, the image association produced by vision is the most prominent. Its formation comes from people’s cognition of products. Products form the language of communication between products and people through their own shaping factors, such as lines, colors, texture, structure, and the connotation given by external cultural environment. The language information conveyed by the product is considered from the perspective of people’s needs. Broadly speaking, any behavior that expresses or communicates thoughts and feelings is called language. Words are silent language, supplemented by gestures, expressions, and movements. Every language has its own vocabulary, called semantics, which combines pronunciation and semantics, and these constitute an infinite semantic field. Semantics means that after thinking, various images are connected with each other, forming a certain ideological relationship, and then generating semantics. As thought is the main content of semantics, it contains images, symbols, concepts, rules, and so on. If the meaning is regarded as a mark or symbol, its connotation refers to the meaning of the meaning, while its external meaning refers to its own meaning conveyed by the meaning. Under the background of the rapid development of Internet economy, how to improve and strengthen the communication and understanding between merchants and consumers; balance the interests of merchants, e-commerce platforms, and consumers; and achieve a win-win situation is particularly important. For e-commerce merchants, the service of merchants is even more important because consumers cannot directly contact and observe products. Before the sale, besides fully conducting market research and designing products according to customers’ needs, it is also necessary to provide detailed product information. Besides providing various data of products in the form of text, it is also possible to provide consumers with more intuitive information through pictures and videos to help them judge. In the past, people made qualitative classification mainly by experience and professional knowledge, which made many classifications subjective and arbitrary, and could not well indicate the intrinsic differences and connections of objective things, especially for the classification of multi-factors and multi-indicators.

Under the influence of consumerism culture, market economy, and business logic have gradually penetrated into the field of mass media. On the one hand, the mass media itself, as the communicator and reflector of society, spreads the values of consumerism. Its communication content has shifted from meaning pursuit to entertainment, constantly creating and stimulating public demand for material consumption and spiritual consumption. On the other hand, with the transformation of society and media, the sources of income of media are diversified. Mass media realize that consumerism can not only meet the needs of the public, but also create greater desire to stimulate the needs of the audience. The consumerism of mass media itself makes it appear the characteristics of money worship in its operation and pursue commercial interests. Therefore, mass media has gradually become a practitioner of consumerism and a typical embodiment of consumer culture.

In order to overcome the deficiency of qualitative classification, it is necessary to introduce mathematical methods and form numerical classification. Data mining, statistics, machine learning, etc., all have research on clustering, and clustering analysis has been applied to many fields. Clustering analysis is to analyze the similarity between pattern vectors corresponding to each sample in the universe, as shown in formula (1).


(1) $$X_{1}\cup X_{2}\cup \ldots \cup X_{N}=X,X_{i}\cap X_{j}=\varphi$$

The membership relationship of sample $X_{k}\left(1\leq k\leq n\right)$ to subset $X_{i}\left(1\leq i\leq c\right)$ can be expressed by membership function as shown in formula (2).


(2) $$u_{x_{1}}\left(x_{k}\right)=u_{k}=1,X_{k}\in X_{t}$$

In the above formula, it is required that every sample is instinctive and can only belong to a certain class, and at the same time, it is required that every subset class is non-empty as shown in formula (3).


(3) $$E_{h}=\left\{u_{k}|u_{k}\in \left\{0,1\right\};\overset{c}{\underset{i=1}{\sum }}u_{k}=1\right\}$$

In the fuzzy clustering division, the sample set X is divided into c fuzzy subsets, and the membership function of the sample is extended from 0,1 binary to the interval $\left[0,1\right]$ as shown in formula (4).


(4) $$E_{f}=\left\{u_{k}|u_{k}\in \left[0,1\right];0<\overset{n}{\underset{k=1}{\sum }}u_{k}<n\right\}$$

Similarity between samples is measured by the distance between samples, such as formulas (5)–(9).


(5) $$d_{ij}\left(1\right)=\overset{m}{\underset{k=1}{\sum }}|X_{ik}-X_{jk}|$$

(6) $$d_{ij}\left(2\right)=\sqrt{\overset{m}{\underset{k=1}{\sum }}\left(X_{k}-X_{jk}\right)}$$

(7) $$d_{ij}\left(q\right)=\left[\overset{m}{\underset{k=1}{\sum }}|X_{ik}-{X_{jk}}^{q}|\right]$$

(8) $$d_{ij}\left(\infty \right)=\max|X_{ik}-X_{jk}|$$

(9) $$d_{ij}\left(M\right)=\left(x_{\left(i\right)}-x_{\left(j\right)}\right)V-1$$

The measure of similarity between attributes can also define the distance, but the similarity coefficient is more commonly used as shown in formula (10).


(10) $$c_{ij}=\frac{\overset{n}{\underset{k=1}{\sum }}x_{ki}}{\sqrt{\left(\overset{n}{\underset{k=1}{\sum }}x^{2}ki\right)}}$$

Through the above algorithm formula, the consumer preference data are analyzed accordingly. When consumers refer to the review information, they should fully consider the characteristics of the product. When consumers choose products, they may ignore some features of products. Therefore, consumers should fully browse the review information, find out the product attributes they are concerned about, and also consider the product features they have not considered.

As a specific manifestation of the trend of consumerism projected into the field of media activities, news consumerism pursues the journalistic concept of audience supremacy, and everything is aimed at the desires of the audience. News media has become the marketer of information products, and the consumability of news at one time has become the fundamental standard of news communication. At the same time, the news intentionally or unintentionally promotes consumerism, emphasizing the importance of material and consumer culture. And the audience itself is also consumed by news, becoming the main actor and participant of news. The media use their own audience resources to plan the prosperity of news.

Under the tendency of news consumerism, the commodity attribute and interest in news are constantly amplified, which is accompanied by the development process of the whole society. Trapping the audience in the symbol game changes the way of thinking and cognitive ability of the public. The social responsibility and professionalism of the media are impacted, and the phenomenon of social alienation is more serious. In essence, the tendency of news consumerism is a kind of control of consumption ideology, and the market-oriented business logic has become the internal driving force of news communication activities. The collision between consumerism and news production makes the spiritual production of news communication and economic material production permeate and cross each other.

## Research on news media marketing and consumer characteristics

### Changes in the characteristics of news consumerism

Among the specific manifestations of news consumerism, the most typical characteristics of news consumerism are the image of the media subject and the communication content of the media. With the progress of science and technology and the transformation of media, journalism is also facing great challenges and changes. In the era of new media, consumerism has produced a new collision in the production and operation of news. News consumerism has added new features to its original features. In the past production society, most of the main images created by the media came from “Heroes.” Through the shaping and publicity of the media, these characters encouraged people to work hard and forge ahead, and played an educational role in citizens. When consumption becomes the center of daily life, various film and television stars and sports stars gradually replace the original “Heroes,” and the audience is also more keen to understand the contents of private life such as family and love behind public figures. These contents bring them a sense of aesthetic pleasure and “gossip” psychological satisfaction, so the media pay more attention to mining the private lives of those idols who are loved by people. Therefore, in the environment of consumerism, “Heroes enlighten readers” has changed into “idols are consumed by readers.” The process of media reporting on these idols has changed into the consumption of their self-image and personal life, realizing spiritual carnival. Influenced by news consumerism, the communication content of news media is more “people-friendly,” which is mainly manifested in the diversification of reporting topics, the storytelling of writing, and the entertainment of news. Under the influence of consumerism, the content of media reports is no longer limited to serious political reports, but changes the communication focus from production mode to lifestyle, and the content of leisure and entertainment, shopping, and travel gradually increases, so as to stimulate consumers’ desire and promote consumption. At the same time, news writing began to pursue storytelling and infectivity, strengthen suspense or sensationalism, enhance the readability of news reports, set news “selling points,” and news became an emotional consumption. In order to meet the needs of the audience, the media itself tends to be entertaining in its reports, and even spoof classic culture, transforming the original “high platform education” into the communication voice of entertaining the public. In order to serve the sensory enjoyment of the audience, a large number of entertainment news and programs appear for the entertainment and enjoyment of the audience, showing an obvious tendency of entertaining the media and culture.

**Figure 4:**
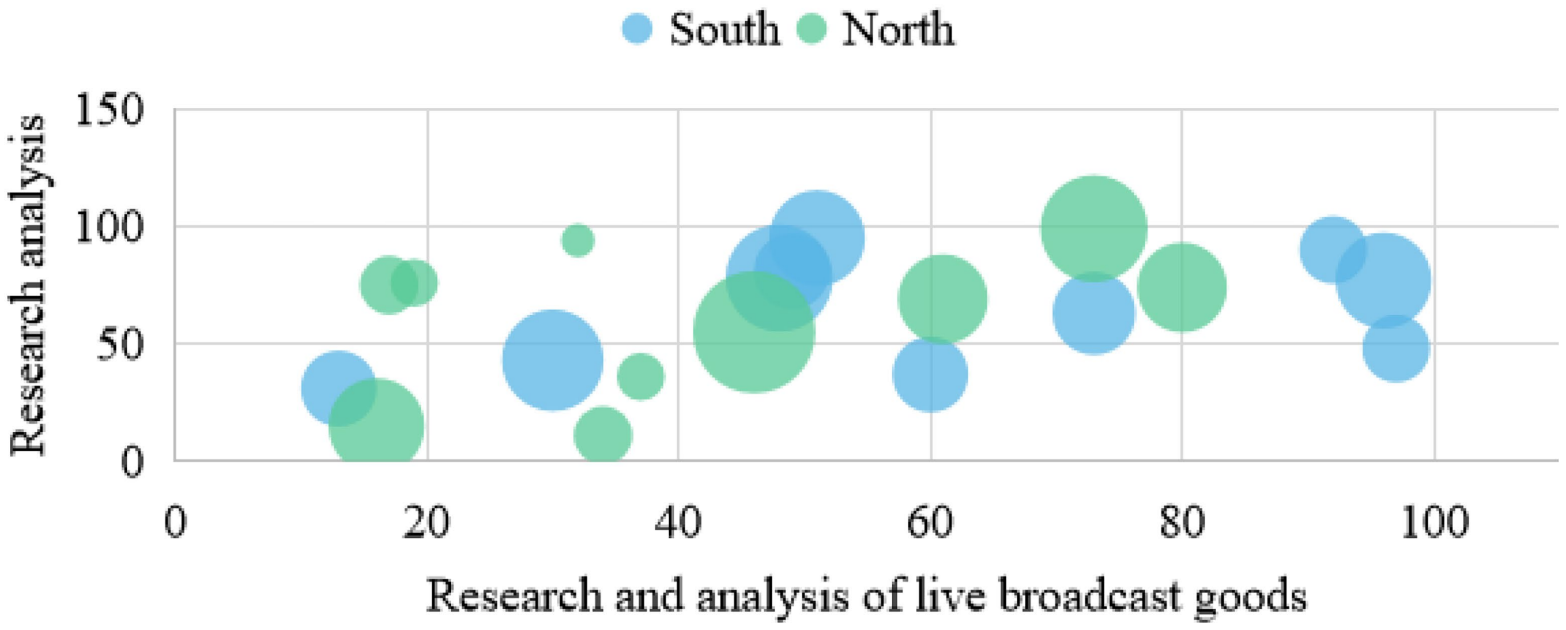
Research and analysis diagram of heat of live broadcast goods.

**Figure 5:**
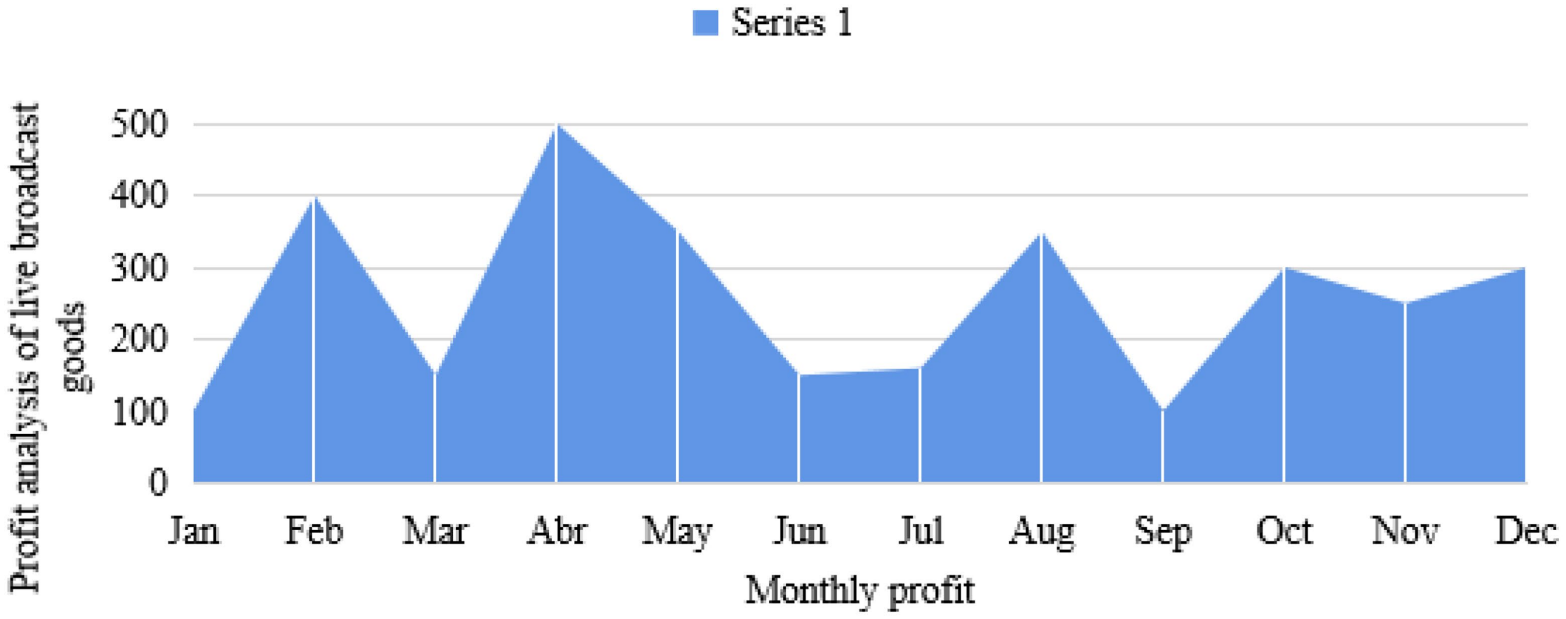
Data analysis of live marketing with goods.

**Figure 6:**
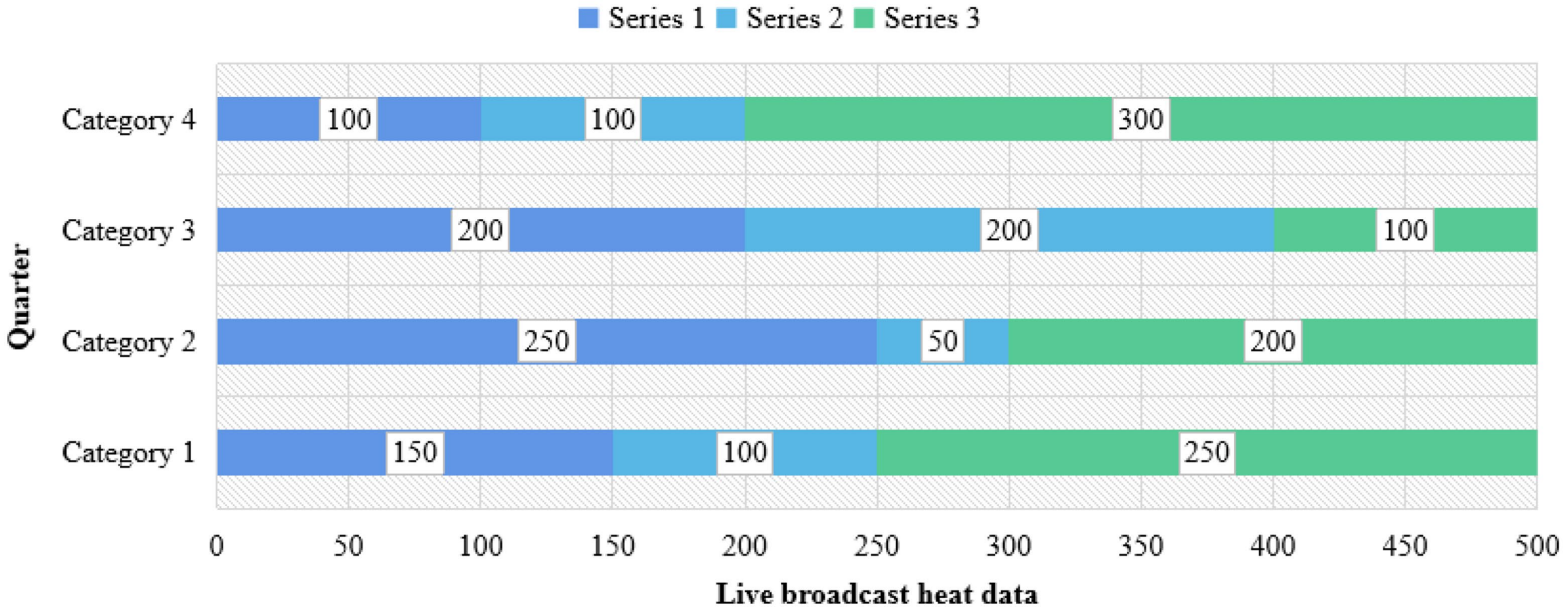
Data map of live delivery marketing efficiency.

News media platforms not only gather a number of groups with purchasing potential, but also expand the social and communication boundaries of evaluating anchors based on the development of live broadcast technology. It can fully realize timely, one to many high-intensity interactions, and jointly create value. Therefore, in the research of commodity live broadcast, relevant data charts are established to analyze and understand them, as shown in Figures 4–6.

Figure 5 shows that the new live marketing with goods is much higher than the traditional marketing, up to about 50.42%. With the evolution of Internet technology and the exploration of digital marketing, live delivery of goods has become a new outlet for retail e-commerce, but the influencing factors of purchasing decisions in its marketing process have always been an unsolved mystery. Environmental factors the marketing environment and economic prospects have a great impact on the development of enterprises, and will inevitably affect their procurement plans. Organizational factors each purchasing organization has its specific objectives, policies, procedures, organizational structure, and systems. The personality and emotion of the specific personnel who carry out the organization’s procurement tasks also play an important role in making the corresponding procurement decisions. Therefore, it is very important to pay attention to the study of personal factors in organizational purchasing behavior and carry out relevant marketing activities with a targeted aim. In view of the above new topic, based on the reality and literature basis of the weakening of traditional purchasing decision factors such as individuals and society, this paper tries to build a model of influencing factors of fans’ purchasing decision on the basis of analyzing the purchasing behavior of live fans with goods, and obtain large sample data by questionnaire survey, so as to empirically investigate the influencing mechanism of platform fans’ purchasing decision under the mode of live marketing with goods. The huge commodity impact brought by the times and material abundance makes it difficult and energy-consuming to choose suitable commodities. Live delivery, a leisure form that combines emotional communication with consumer persuasion, relaxation, and sharing good things, meets the needs of users. In the early e-commerce live broadcast, only one anchor appeared, and introduced the functions of the products. The content of the live broadcast was relatively simple, and more focused on the practical and rational utilitarian value, similar to the hard and wide form in advertising. Live broadcast breaks through the blunt sales form, weakens the capital attribute of live broadcast, reduces the function of transmitting commodity information, and shows an obvious trend of pan-entertainment. Quality is the life of an enterprise, the life of a product, and the foundation of live promotion. Therefore, every node of the product supply chain with live goods should strictly control the quality, and a full traceability system of product circulation should be established on a more scientific basis, so that the whole process of product circulation can be traced, so as to ensure that consumers’ demands can be better met. Social division of labor has brought about the improvement of production efficiency, but it has also caused the separation of production and consumption and the information asymmetry between producers and consumers. The core value of live delivery and traditional sales promotion lies in providing services to eliminate information asymmetry between production and consumption, so as to increase the overall welfare of society.

### Research on the anchor staff under live broadcast with goods

**Figure 7:**
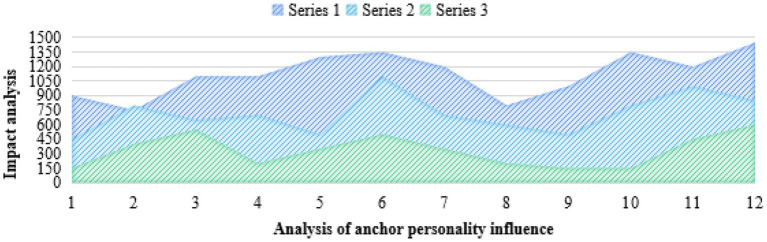
Analysis of the influence of anchor personality.

**Figure 8:**
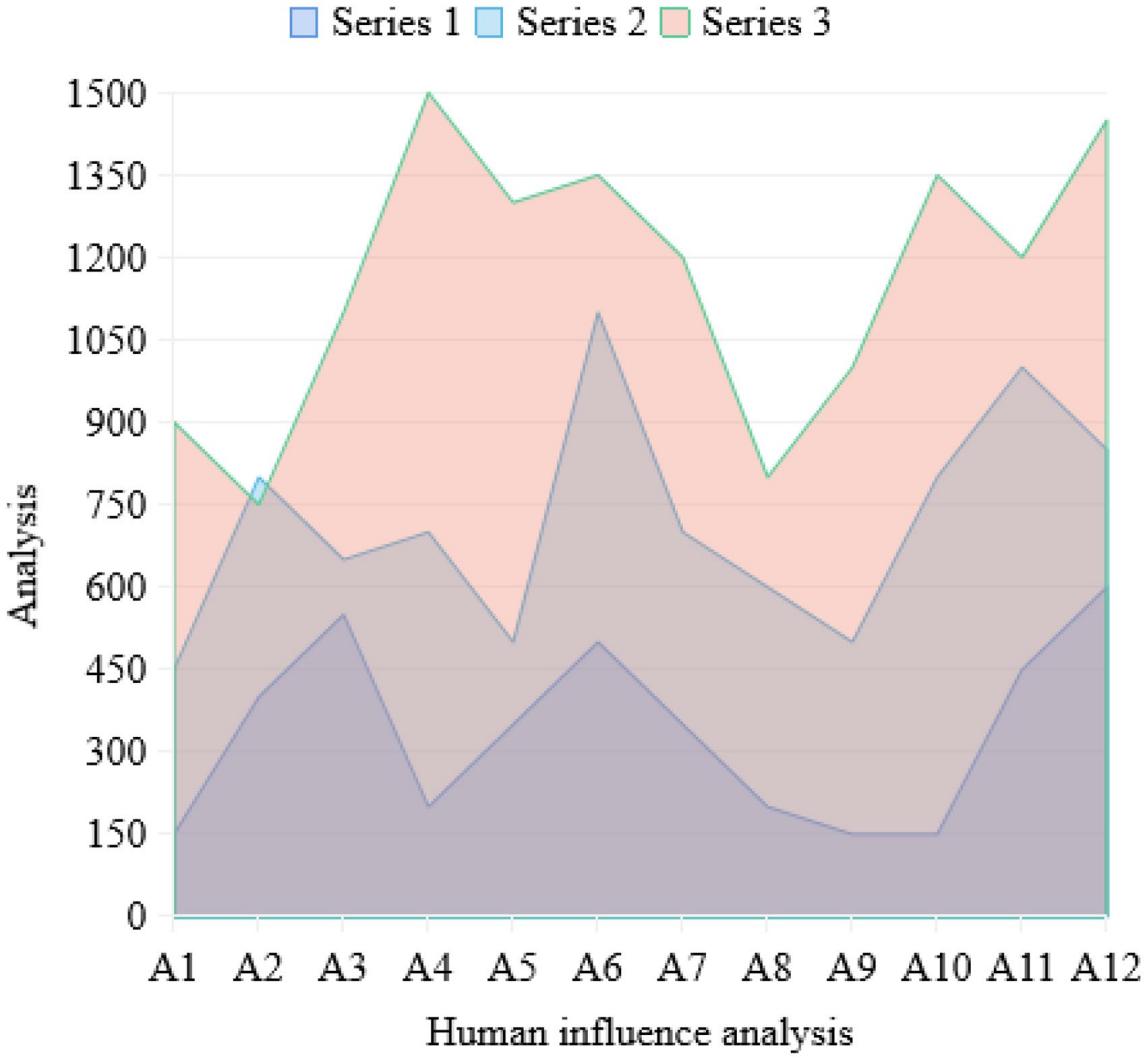
Data analysis diagram of anchor personnel.

The companionship of the live broadcast is also reflected in the fact that users can participate in the live broadcast interaction at any time through likes, comments, barrage, grabbing coupons, etc., and get a sense of synchronization, immediacy, and presence. Fans in the live broadcast area form a companion social interaction due to the interaction caused by watching together. At present, social mobility and social support alienation easily lead to loneliness. Enjoying company in the lively live broadcast room of the anchors essentially shows that atomized individuals can get emotional resonance and spiritual sustenance in participating in e-commerce consumption interaction. For users, live delivery is not only a way of consumption to improve the quality of life by consuming goods, but also an emotional decompression valve to relieve the pressure of life, and an emotional companion to obtain social support and relieve loneliness. Consumers are often imaginative, eager for change, like innovation, have strong curiosity, and put forward higher requirements for personalized consumption. What they choose is not only the practical value of commodities, but also to be different and fully reflect their own value, which has become the primary standard of their consumption. The unique shopping environment and the different purchasing methods from the traditional trading process will cause consumers’ curiosity, detachment, and personal emotional changes. In this way, consumers can challenge merchants according to their own wishes, take themselves as the center, act according to their own ideas, and fully express themselves in consumption. Person design refers to the setting of characters. It was originally used as a professional vocabulary in cartoons, animations, and movies, including the personality characteristics, appearance characteristics, and character modeling set for a certain character. In recent years, it has been frequently used to shape the public image of stars. The reason is that the labels given to stars by people can target more accurate people and enhance the commercial value of stars. In the face of such a live broadcast anchor who pursues attention and product conversion rate, the shaping of people is equally important. There are some similarities between the network anchor and the host of traditional entertainment programs in form. The network anchor can be regarded as the host of a self-media program. Both of them have the same pursuit of personalized communication. The host often leaves more impression on the audience than the program itself. The overall effect of the product can be improved through the host’s explanation of the product. However, this requires the moderator to respect the objective facts and ensure the authenticity when explaining the products. Do not exaggerate so as to mislead the audience. The common language also makes the host’s own hosting style. Use the method of “story telling” to make the product language concise and clear, and increase the sense of intimacy. They both hope that in the process of communication, through personality charm, language behavior, and other ways, the audience will feel friendly and human, and they will resonate with the audience. Idealized performance refers to the performance that strictly follows the accepted social norms or the objective social expectations. For the anchor with goods, the idealization of performance means giving the audience a perfect “performance.” In the early stage, the anchor with goods should choose the product category that is most suitable for the audience according to the field they specialize in, and strive to bring the most favorable price to consumers. In the live broadcast, the “stage” of the live broadcast room should be carefully arranged, the truest state of the product should be restored through suitable lighting, the main selling points of the product and the applicable people should be concisely expressed, and consumers’ feelings after using the product should be told through personal experience. All kinds of behaviors of anchors in the dissemination of content may affect the audience’s thoughts and behaviors. Therefore, the behavior of the anchor with goods in the live broadcast room should also follow the norms and must not lead to misleading behavior. In the research, the corresponding data graphs are established as shown in Figures 7, 8.

In Figure 7, it can be seen that the personality of the anchor has a great relationship with the consumption degree of consumers and the sales rate of products, and the influence is as high as 78.53%. Idealization does not mean losing authenticity. If you give yourself too many beauty filters in the shaping of people’s designs, you will face the crisis of “collapse of people’s designs.” The introduction of products must not be falsified, and there should be no unfounded boasting in order to increase sales. Distorted idealized performance will reduce the public trust and goodwill of the anchor. At the moment when live marketing with goods is in full swing, the phenomenon of online false marketing and selling fake goods occurs from time to time. As a marketing target, platform fans should improve their discrimination ability, and rationally choose and buy anchor recommendations. Mysterious performance refers to the performance that arouses and maintains the audience’s awe by keeping social distance. Moderate background content foreground can narrow the distance between media and audience, but excessive exposure of background content also faces the risk of “collapse of people.” Backstage mystification can relieve the pressure of the anchor to a certain extent and stimulate the curiosity of the audience.

## Conclusion

In the tendency of media consumerism, the consumability of media is the most fundamental standard. In the new media era, news consumption has gradually become a normal. In the process of transformation and integration, traditional media continue to innovate the means of news production. The emergence of paid news, live broadcast, and other media forms has led to the emergence of more news products. News itself has really become a product that can produce economic value. It has appeared in public view and become a daily consumer good. In the Internet era, the commercial marketing mode of live media has become an important representative of modern marketing mode. And will flourish with the further development of network economy. Studying the basic characteristics of the phenomenon live broadcast commodity marketing model has important theoretical value for analyzing the live broadcast commodity marketing model. It also points out the direction for the future scientific, healthy, and sustainable development of live broadcast commodity marketing mode. As an emerging profession, network hosts are currently facing many problems. In addition to the immature external environment and non-standard career development, the host’s own comprehensive quality is also its biggest deficiency. Just as consumerism plays a role in promoting the economy in social development, news consumerism will also play a positive role in the reform of news production technology. In order to better attract readers, win the target audience, and meet the audience’s needs for future news production. News consumerism makes the media take the initiative to meet the challenges of the new media era. Actively carry out transformation and integration, adapt to the changes of the times, innovate the technological changes of news production, and enhance their competitiveness with better news products. The study has some limitations. The proposed method lacks the validation of case data. The validity and feasibility of this method cannot be proved. This paper also needs to further prospect the application of data mining technology in consumer behavior research.

## Data availability statement

The original contributions presented in the study are included in the article/supplementary material, further inquiries can be directed to the corresponding author.

## Author contributions

All authors listed have made a substantial, direct, and intellectual contribution to the work and approved it for publication.

## Conflict of interest

The authors declare that the research was conducted in the absence of any commercial or financial relationships that could be construed as a potential conflict of interest.

## Publisher’s note

All claims expressed in this article are solely those of the authors and do not necessarily represent those of their affiliated organizations, or those of the publisher, the editors and the reviewers. Any product that may be evaluated in this article, or claim that may be made by its manufacturer, is not guaranteed or endorsed by the publisher.
